# Removal of B from Si by Hf addition during Al–Si solvent refining process

**DOI:** 10.1080/14686996.2016.1140303

**Published:** 2016-02-23

**Authors:** Yun Lei, Wenhui Ma, Luen Sun, Jijun Wu, Yongnian Dai, Kazuki Morita

**Affiliations:** ^a^ State Key Laboratory of Complex Nonferrous Metal Resources Clean Utilization, Kunming University of Science and Technology, Kunming650093, PR China;; ^b^ National Engineering Laboratory for Vacuum Metallurgy, Kunming University of Science and Technology, Kunming650093, PR China;; ^c^ Department of Materials Engineering, Graduate School of Engineering, The University of Tokyo, 7-3-1 Hongo, Bunkyo-ku, 113-8656Tokyo, Japan

**Keywords:** Electromagnetic solidification refining, Al–Si solvent, silicon purification, hafnium addition, boron removal, 10 Engineering and structural materials, 106 metallic materials, 503 TEM, STEM, SEM, 301 chemical syntheses/processing

## Abstract

A small amount of Hf was employed as a new additive to improve B removal in the electromagnetic solidification refinement of Si with an Al–Si melt, because Hf has a very strong affinity for B. The segregation ratio of Hf between the solid Si and Al–Si melt was estimated to range from 4.9 × 10^−6^ to 8.8 × 10^−7^ for Al concentrations of 0 to 64 at.%, respectively. The activity coefficient of Hf in solid Si at its infinite dilution was also estimated. A small addition of Hf (<1025 parts per million atoms, ppma) significantly improved the B removal. It was confirmed that the use of an increased Hf addition, slower cooling rate, and Al-rich Al–Si melt as the refining solvent removed B more efficiently. B in Si could be removed as much as 98.2% with 410 ppma Hf addition when the liquidus temperature of the Al–Si melt was 1173 K and the cooling rate was 4.5–7.6 K min^–1^. The B content in Si could be controlled from 153 ppma to 2.7 ppma, which meets the acceptable level for solar-grade Si.

## Graphical abstract

**Figure:**
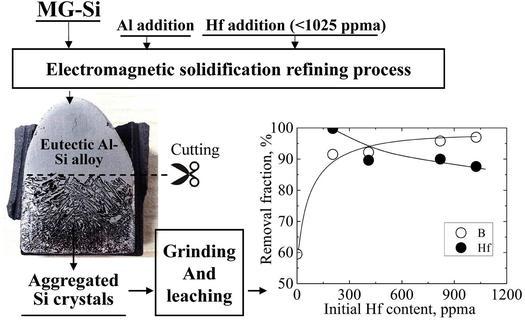

## 1. Introduction

Reducing the cost of producing solar-grade Si (SoG-Si) is important for manufacturing low-cost Si solar cells. One promising method involves upgrading metallurgical-grade Si (MG-Si) to SoG-Si with metallurgical treatment. Boron, an impurity in MG-Si, cannot be easily removed through solidification refining and vacuum melting because of its large segregation coefficient (0.8 at 1687 K) [1] and low vapor pressure (lower than that of Si) [2]. Some processes such as oxidation through H_2_O-added plasma melting [3, 4] and slag treatment [5–8] are currently employed for B removal. However, a further reduction in cost and a more environmentally friendly refining process for manufacturing SoG-Si are still needed.

A solvent-refining process using an Al–Si melt combined with electromagnetic solidification yielded outstanding results in purifying Si more effectively and economically [9, 10]. The Si crystals can be purified because of the low segregation ratios of the impurities, and they can be simultaneously separated from the Al–Si solvent with electromagnetic force. Some attempts have also been made to improve B removal and Si separation based on this solvent-refining process, such as using Si–Al–Sn (10–30 mol%) [11] and Si–Al–Zn (<40 mol%) [12] melts as the refining solvents and improving the electromagnetic stirring [13, 14]; however, further efforts are necessary to control the concentration of residual B to an acceptable level (< 2.6 ppma or 1 ppmw, where ppma and ppmw stand for parts per million by atom and weight, respectively) for producing SoG-Si. Adding small amounts of special additives might improve the B removal process, controlling the concentration of residual B in Si to an acceptable level. A small amount of Ti has been used as an additive to improve B removal because B and Ti can form a thermodynamically stable compound, TiB_2_ [15]. The B content of refined Si was reduced from 170 to 1.1 ppma by adding 933 ppma Ti, and the added Ti could be simultaneously reduced from 933 to 1.6 ppma. In a previous study [16], we added a small amount of Zr (<1057 ppma) to improve B removal in the electromagnetic solidification refining process.

In this study, we attempted to use Hf as a new additive to improve B removal for two reasons: (1) HfB_2_ is the most stable boride based on the author’s knowledge. Therefore, Hf has a stronger affinity for B than Al, Si, and Ti [17, 18], which implies that the addition of Hf is potentially more beneficial to B removal than that of Ti. (2) The segregation coefficient of Hf was estimated to be very small in this study, which implies that the added Hf could be simultaneously removed during the solvent refining. Even though there might be some residual Hf in Si, it can be further removed in the following directional-solidification refining process and will not contaminate the Si crystals. Therefore, B removal by Hf addition in electromagnetic solidification refining of Si with an Al–Si melt was investigated in this study.

## 2. Experimental details

### 2.1. Segregation ratio of Hf between solid Si and Al–Si melt

The chemical potential of Hf is identical between solid Si and the Al–Si melt during the solidification refining process, which can be expressed by Equation (1):

(1) $$\mu_{\text{Hf(l) in Al - Si melt}}^{\circ }+\text{R}T\ln a_{\text{Hf(l) in Al - Si melt}}=\mu_{\text{Hf(s) in solid Si}\;}^{\circ }+\text{R}T\ln a_{\text{Hf(s) in solid Si}}$$

where $\mu_{Hf}^{{}^{\circ }}$ and aHf are the chemical potential of Hf and activity of Hf at its standard state, respectively. The characters l and s in parentheses denote the liquid and solid standard state, respectively. *a*_*Hf*_ is assumed to obey Henry’s law as the Hf content in both phases is very small (<1025 ppma). The segregation ratio of Hf between solid Si and the Al–Si melt at its infinite dilution, $k_{Hf}^{{}^{\circ }}$, can be expressed by Equation (2):

(2) $$\ln k_{\text{Hf}}^{\text{o}}=\ln\frac{X_{\text{Hf in solid Si}}}{X_{\text{Hf in Al - Si melt}}}=\frac{\Delta G_{\text{Hf}}^{\text{fus}}}{RT}+\ln\frac{\gamma_{\text{Hf (l) in Al - Si melt}}^{\circ }}{\gamma_{\text{Hf (s) in solid Si}}^{\circ }},$$

where $X_{\text{Hf}}$, $\Delta G_{\text{Hf}}^{\text{fus}}$, and $\gamma_{\text{Hf}}^{\text{o}}$ are the mole fraction, standard Gibbs energy change for fusion, and activity coefficient at the infinite dilution of Hf, respectively. $\gamma_{\text{Hf (l) in Al - Si melt}}^{\circ }$ can be calculated using Equation (3) by applying the Gibbs–Duhem integration method developed by Toop [19].

(3) $$\begin{array}{c} \text{R}T\ln\gamma_{\text{Hf (l) in Al - Si melt}}^{\circ }=X_{\text{Si in Al - Si melt}}\text{R}T\ln\gamma_{\text{Hf (l) in molten Si}}^{\circ }\\ \;+X_{\text{Al in Al - Si melt}}\text{R}T\ln\gamma_{\text{Hf (l) in molten Al}}^{\circ }\\ \;\text{-}\;\Delta G_{\text{in Al - Si melt}}^{\text{M, ex}} \end{array},$$

where $\gamma_{\text{Hf (l) in molten Si}}^{\circ }$ and $\gamma_{\text{Hf (l) in molten Al}}^{\circ }$are treated in Equations (4) and (5), respectively.

(4) $$RT\ln\gamma_{\text{Hf (l) in molten Si}\;}^{\circ }={\left[\Delta G_{\text{in Si - Hf melt}}^{\text{M, ex}}-X_{\text{Si}}\frac{\partial \Delta G_{\text{in Si - Hf melt}}^{\text{M, ex}}}{\partial X_{\text{Si}}}\right]}_{X_{\text{Si}}=1}$$

(5) $$RT\ln\gamma_{\text{Hf (l) in molten Al}\;}^{\circ }={\left[\Delta G_{\text{in Al - Hf melt}}^{\text{M, ex}}-X_{\text{Al}}\frac{\partial \Delta G_{\text{in Al - Hf melt}}^{\text{M, ex}}}{\partial X_{\text{Al}}}\right]}_{X_{\text{Al}}=1}$$

$\Delta G_{\text{in A - B melt}}^{\text{M, ex}}$ in Equations (3)–(5) denotes the excess Gibbs energy for mixing of A and B, which can be treated in Equation (6) by applying the Redlich–Kister type regular solution model.

(6) $$\Delta G_{\text{in A - B melt}}^{\text{M,ex}}=\sum_{i=0}^{j}\Omega_{\text{A - B}}^{i}{\left(X_{\text{A}}-X_{\text{B}}\right)}^{i}X_{\text{A}}^{}X_{\text{B}}$$

According to the phase diagram of the Hf–Si system [20], the Si solid solution is in equilibrium with the intermediate phase, HfSi_2_, below 1603 K. Its formation reaction is written as Equation (7), and the standard Gibbs energy for this reaction can be expressed by Equation (8).

(7) $$\text{Hf}\;\left(\text{s}\right)+\;\text{2Si}\;\left(\text{s}\right)\;={\;\text{HfSi}}_{\text{2}}\left(\text{s}\right)$$

(8) $$\Delta G^{\circ }=-209100+31.4T\left(J/mol\right)\left[17\right]$$

Because the solubility of Hf in solid Si is very small (less than 0.08 ppma below 1373 K [21]), the activity coefficient of Hf in solid Si was assumed to obey Henry’s law. $\gamma_{\text{Hf (s) in solid Si}}^{\circ }$ can be estimated using Equation (9):

(9) $$\Delta G^{\circ }=-RT\frac{\ln a_{{\text{HfSi}}_{\text{2}}\;\text{(s)}}}{a_{\text{Hf (s)}}a_{\text{Si}}^{\text{2}}}≅RT\ln\gamma_{\text{Hf (s) in solid Si}}^{\circ }X_{\text{Hf (s) in solid Si}}^{*}{\left(1-X_{\text{Hf (s) in solid Si}}^{*}\right)}^{2},$$

where $X_{\text{Hf (s) in solid Si}}^{*}$ is the solubility of Hf in solid Si.

### 2.2. Solidification of Al––Si melt with electromagnetic force

**Figure 1.**
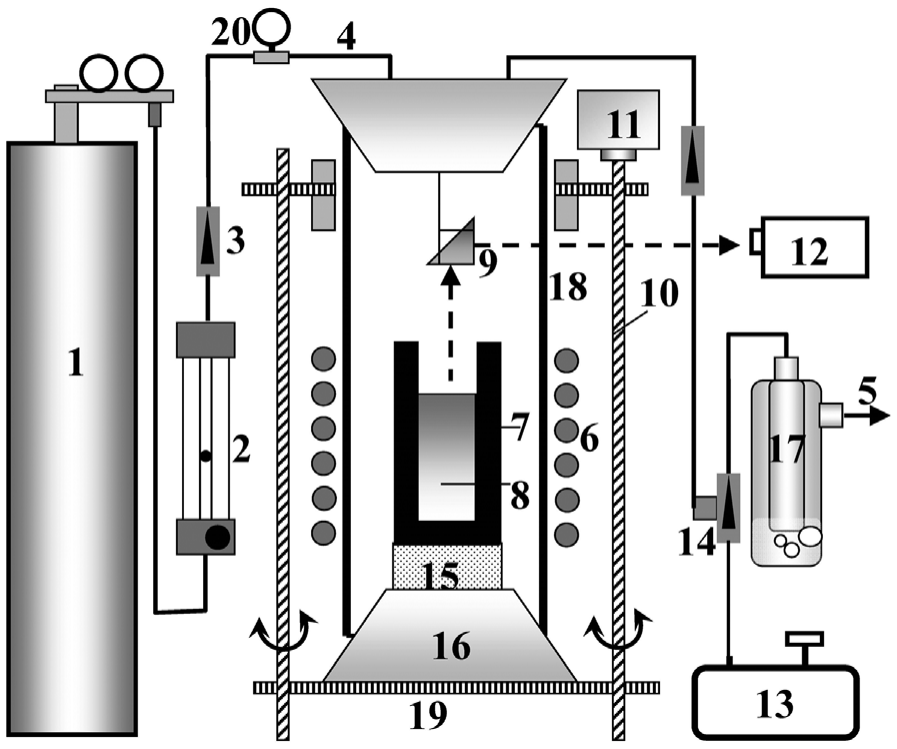
Schematic of the experimental apparatus: 1, Ar gas (99.99%) tank; 2, gas flow meter; 3, two-way valve; 4, gas inlet; 5, gas outlet; 6, induction coils; 7, graphite crucible; 8, Al–Si melt; 9, prism; 10, ball screw; 11, stepping motor; 12, infrared pyrometer; 13, vacuum pump; 14, three-way valve; 15, porous alumina holder; 16, silicone plug; 17, bubble checking; 18, quartz chamber; 19, stainless steel plate; 20, vacuum meter.

Ten grams of bulk Si (99.9999%) and Al shot (99.999%) together with different amounts of Si–1 wt% B and Si–10 wt% Hf alloys were placed in a high-purity dense graphite crucible (25 mm outer diameter, 17 mm inner diameter, 65 mm length). The bottom of the crucible was placed in level with the lower end of the induction coils, as shown in Figure 1. The Si–1 wt% B and Si–10 wt% Hf alloys were prepared by melting bulk Si with B powder (99.9%) and Hf lump (99.5%), respectively, and subsequently grinding the mixtures into powders (particle diameter < 186 μm) in an agate mortar to homogenize their compositions. The compositions of the Si–1 wt% B and Si–10 wt% Hf alloys were confirmed by inductively coupled plasma-atomic emission spectroscopy (ICP-AES). After melting and holding at 1473 K for 30 min, the sample was cooled by lowering the crucible at a constant rate for solidification refining.

The high-Si-density region of the solidified sample was removed from the Al–Si alloy for analysis. One part was subjected to scanning electron microscopy (SEM) analysis coupled with energy dispersive X-ray spectroscopy (EDS). The remaining part was crushed into a powder (particle diameter < 186 μm) and treated with aqua regia containing H_2_SO_4_ (HCl:HNO_3_:H_2_SO_4_ = 3:1:1) for 6 h to remove B, Hf, and Al in the intergranular and liquid phases. The acid-treated Si powder was then dissolved using a mixed acid comprising HNO_3_, HF, H_3_PO_4_, and H_2_SO_4_ (HNO_3_: H_3_PO_4_:H_2_SO_4_ = 10:1:1, addition of HF drop by drop) to analyze its chemical composition using ICP-AES.

## 3. Results and discussion

### 3.1. Segregation ratio of Hf between solid Si and Al–Si melt

$\gamma_{\text{Hf (l) in Al - Si melt}}^{\circ }$in Equation (2) can be calculated using Equations (3)–(6). The parameters used for the calculation are listed in Table 1 [20, 22, 23].

**Table 1.** Parameter sets for Al–Si, Si–Hf and Al–Hf systems.

System	Regular solution parameter, J/mol
Al–Si [22]	$\Omega_{\text{Al - Si}}^{0}=-10695.4-1.823T$
	$\Omega_{\text{Al - Si}}^{1}=-4274.5+3.044T$
	$\Omega_{\text{Al - Si}}^{2}=670.7-0.46T$
Si–Hf [20]	$\Omega_{\text{Si - Hf}}^{0}=-177631+6.42546T$
	$\Omega_{\text{Si - Hf}}^{1}=-1830$
Al–Hf [23]	$\Omega_{\text{Al - Hf}}^{0}=-182442+22.138T$
	$\Omega_{\text{Al - Hf}}^{1}=-21934+20.534T$
	$\Omega_{\text{Al - Hf}}^{2}=54575$

The solubility of Hf in solid Si was reported for the temperature range of 1373–1523 K by Sachdeva et al*.* [21]. The data for the interstitial solution was employed as the low limit of its solubility in solid Si. $RT\ln\gamma_{\text{Hf (s) in solid Si}}^{\circ }$was estimated using Equation (9) and was expressed as Equation (10).

(10) $$RT\ln\gamma_{\text{Hf (s) in solid Si}}^{\circ }=61110\left(\pm 16200\right)-30\left(\pm 11\right)T\left(J/mol\right)$$

The estimated segregation ratios of Hf between solid Si and the Al–Si melt at its infinite dilution are listed in Table 2. The segregation coefficient of Hf between solid Si and liquid Si at its infinite dilution (at the melting point of Si, 1687 K) was estimated to be 4.9 × ×10^−6^.

**Table 2.** Estimated segregation ratios of Hf between solid Si and Al–Si melt at its infinite dilution at different liquidus temperatures.

Temperature (K)	Segregation ratio
1687	4.9×10^−6^
1573	7.1×10^−6^
1473	6.2×10^−6^
1373	4.4×10^−6^
1273	2.3×10^−6^
1173	8.8 ×10^−7^

### 3.2. Solidification of Al–Si melt with electromagnetic force

The initial composition of the Al–Si melt and the final composition of refined Si are listed in Table 3. The B content in typical MG-Si is reported to be between 13 and 130 ppma [24]. However, the initial B content of the Al–Si melt in this study was set to 153 ppma (60 ppmw), considering possible contamination from Al in the practical refining process. The initial B and Hf contents of the Al–Si melt were controlled by precisely weighing the powdered Si–1 wt% B and Si–10 wt% Hf alloys, respectively.

**Table 3.** Initial composition of Al–Si melt and final composition of refined Si with different lowering rates.

No.	Loweringrate, mm min^–1^ (±0.02)	Initial composition of the Al–Si melt			Final composition of refined Si, ppma		
		B, ppma	Hf, ppma	Al, at.%	B	Hf	Al
1	0.55	153	0	55.0	62.0	0	317
2	0.55	153	205	55.0	13.0	0.4	439
3	0.55	153	410	55.0	12.0	42.6	496
4	0.55	153	820	55.0	6.4	82.0	526
5	0.55	153	1025	55.0	4.6	127	435
6	0.55	153	410	64.0	2.7	66.8	611
7	0.55	153	410	45.0	37.3	39.4	473
8	0.22	153	410	55.0	10.6	26.1	353
9	0.41	153	410	55.0	12.8	39.4	401
10	1.36	153	410	55.0	17.3	131	848

**Figure 2.**
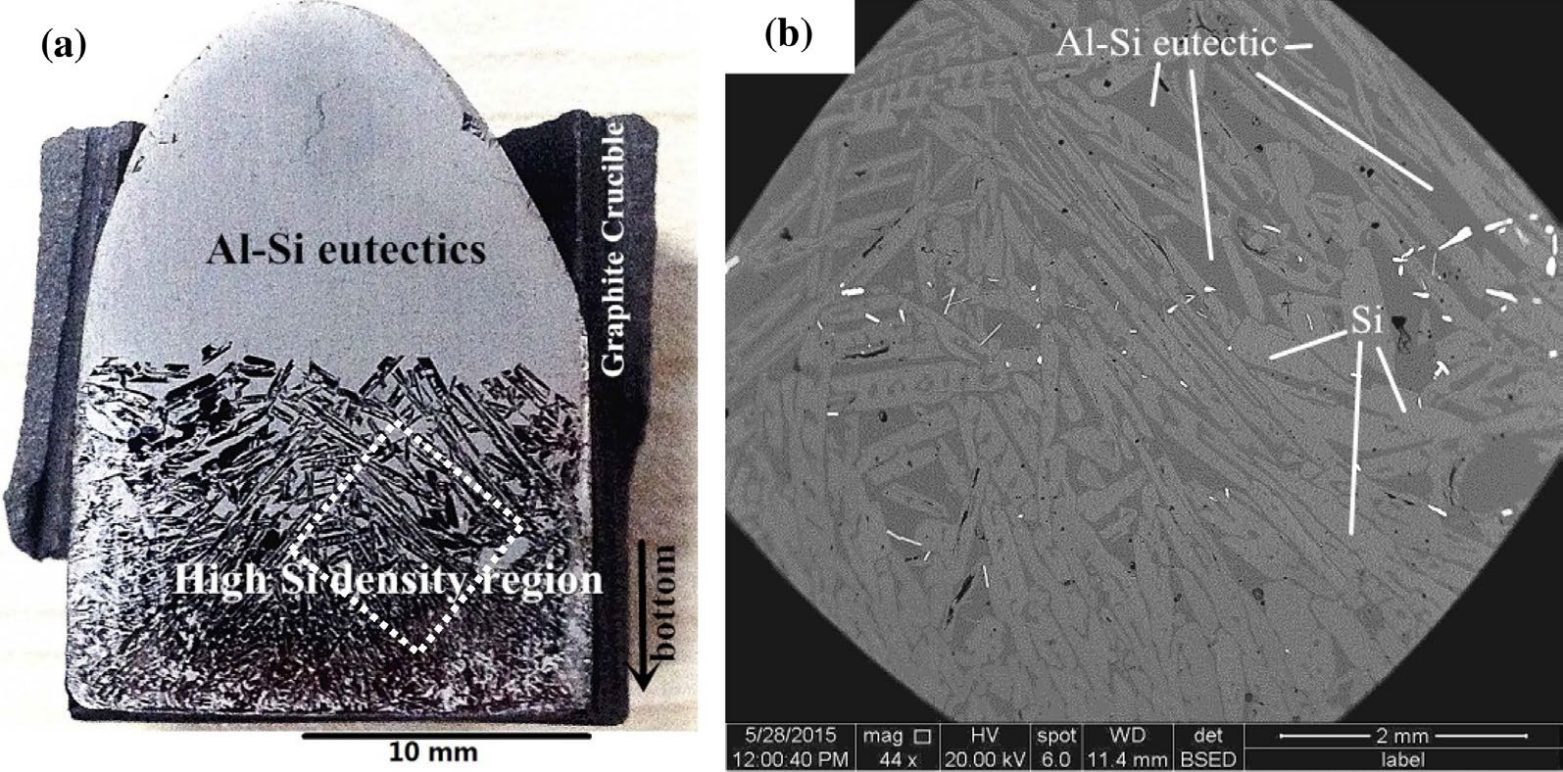
(a) A photograph of the cross-section of an Al–45 at.% Si alloy and (b) a magnified image of the rhombic region in (a).

A cross-section of an alloy (No. 5 in Table 3) solidified with electromagnetic force is shown in Figure 2(a). The image indicates that Si crystals were successfully agglomerated at the bottom of the sample with electromagnetic force, i.e. the primary Si crystals and eutectic Al–Si melt were successfully separated. This phenomenon will significantly reduce the consumption of acid solution in the subsequent leaching process. The mechanism of this agglomeration has been explained in detail in other studies [9, 10, 14]. Figure 2(b) presents a magnified image of the rhombic region in Figure 2(a).

**Figure 3.**
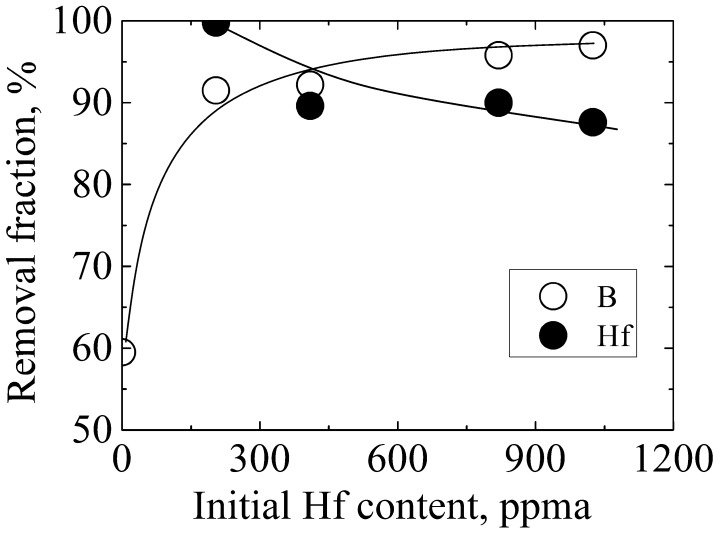
Removal fractions of B and Hf after electromagnetic solidification refinement of Si with varying initial Hf content.

Figure 3 shows the effect of Hf addition on B removal at the lowering rate of 0.55 mm min^–1^. The refining solvent was fixed as Al–45 at.% Si melt. When the Hf content increased from 0 to 1025 ppma, the fraction of B removed increased from 59.5% to 97.0%, indicating that Hf was significantly responsible for the decrease in B content in the refined Si. Simultaneously, the fraction of Hf removed decreased from 99.8% to 87.6% with the increase of its initial content, because it was difficult for a larger amount of Hf to diffuse sufficiently from the Si grains into the intergranular and liquid phases in a limited time.

**Figure 4.**
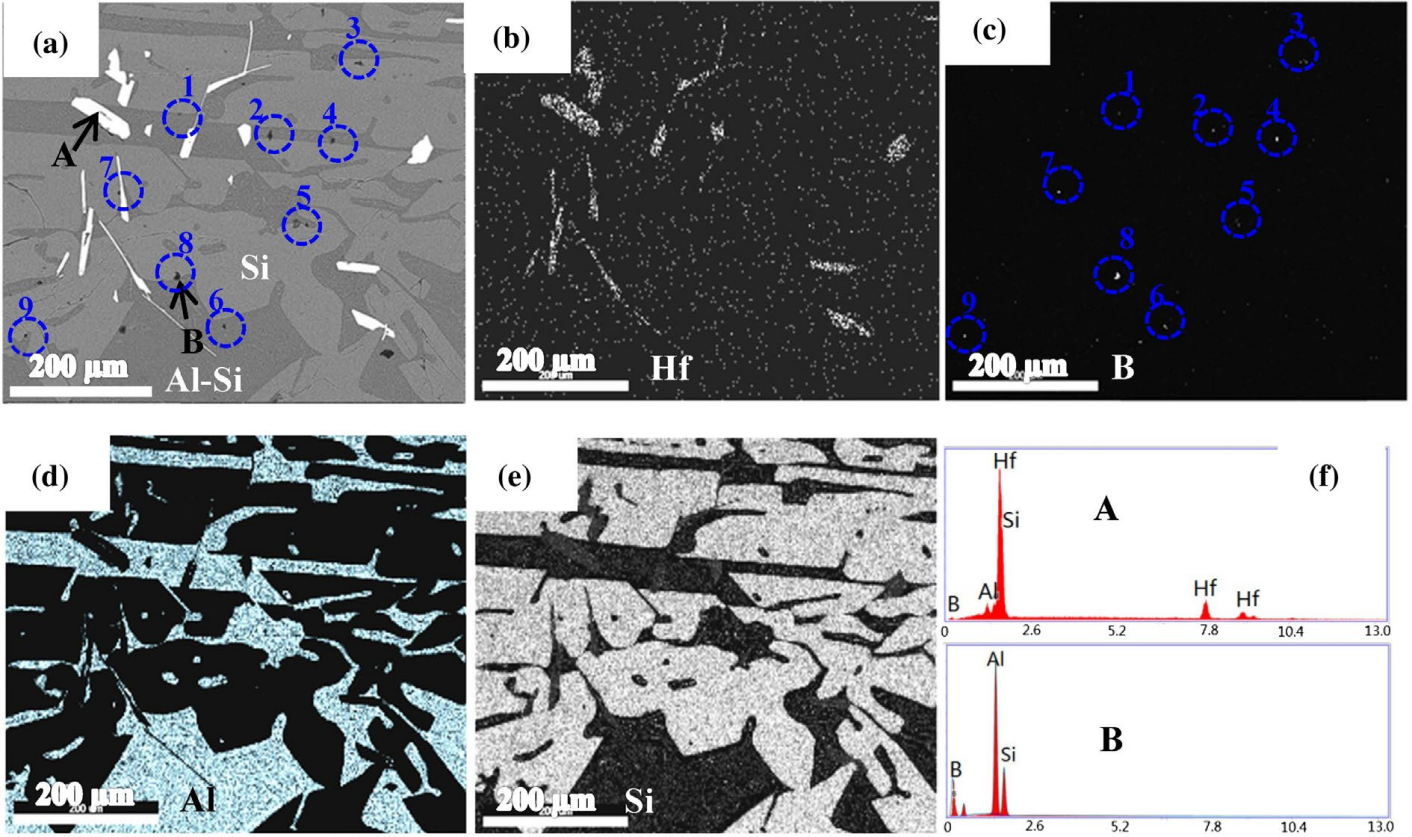
SEM image and EDS analysis of a region in a solidified sample (No. 5 in Table 3): (a) SEM image. EDS maps of (b) Hf, (c) B, (d) Al, and (e) Si. (f) EDS analysis for phases A and B shown in (a). (Blue circles 1–9 indicate the positions of B containing particles 1–9, respectively, the white spot in each blue circle shown in Figure 4(c) indicates the B containing particle).

SEM analysis with EDS was performed to explain the efficient removal of B by Hf addition. Figure 4 presents an SEM image and EDS analysis of a region in a solidified sample (No. 5 in Table 3). Some white phase was observed along the Si grain boundaries and was determined to be an Hf-rich phase (Figure 4(b)). The phase mainly consisted of Hf, Si, and Al with a small amount of B according to the EDS analysis (Figure 4(f)). Qualitative analysis indicated that the mole ratio of Hf, Al, and Si was 1:0.2:1.8. HfAl_0.2_Si_1.8_ is the binary intermetallic compound HfSi_2_ with certain amount of Al solubility. The small amount of B possibly originates from the solid solution. This Hf-containing compound formed along the Si grain boundaries because of the small solubility of Hf in solid Si (less than 0.08 ppma below 1373 K [21]). Thus, the Hf-containing compound can be easily exposed to the leaching acid solution after grinding Si crystals, making Hf most responsive to being removed.

**Figure 5.**
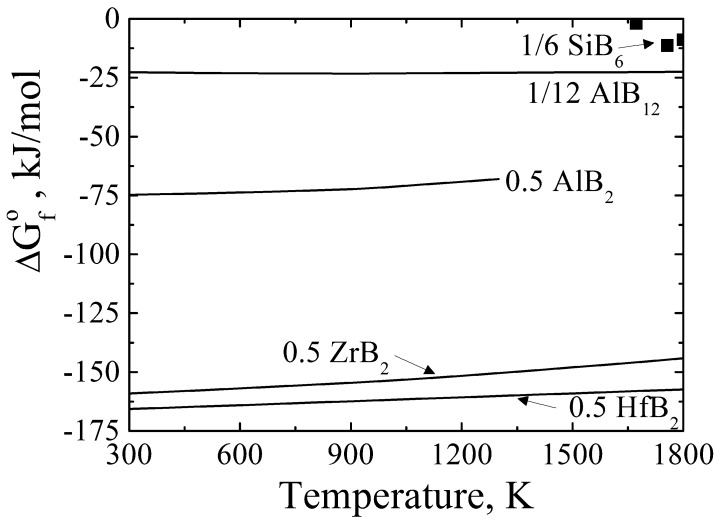
The Ellingham diagram for some borides.

Some black particles were also observed at the Si grain boundary and in the Al–Si phase, which was identified as an Al–Si–B phase by EDS analysis, as shown in Figure 4(c) and (f). The mole ratio of Al and Si was determined to be 3.2:1, which was close to that of the reported solid solution Al_0.77_Si_0.23_B_12_ [25] (qualitative analysis was not performed for B because it could not be determined accurately by EDS). Although HfB_2_ was expected to form as it has a significantly stronger affinity for B than Si and Al, as observed in Figure 5, we could not detect HfB_2_ in this study. The Al–Si solvent was thought to dilute the concentration of Hf and reduce the reaction chance of Hf and B. Similarly in the Hf case, the reason for the efficient B removal could be the B-containing compound, such as the Al–Si–B phase, forming along the Si grain boundaries and in the Al–Si melt, resulting in B removal by acid leaching.

The chemical potential of B in the Al–Si melt and solid Si is identical at equilibrium, as shown in Equation (11). Therefore, the segregation ratio of B between solid Si and Al–Si melt (containing Hf) can be expressed as Equation (13).

(11) $$\mu_{\text{B (in solid Si)}\;}=\mu_{\text{B (in liquid Si)}\;}$$

(12) $$\text{R}T\text{ln}a_{\text{B(s) in solid Si}}=\Delta G_{\text{B}}^{\circ \;\text{fus}}\;\text{+ R}T\text{ln}a_{\text{B(l) in Al - Si melt}}$$

(13) $$\ln k_{\text{B}}\;\text{= ln}\frac{X_{\text{B(s) in solid Si}}}{X_{\text{B(l) in Al - Si melt}}}=\frac{\Delta G_{\text{B}}^{\circ \;\text{fus}}}{\text{R}T}\;\text{+ ln}\frac{\gamma_{\text{B(l) in Al - Si melt}}}{\gamma_{\text{B(s) in solid Si}}}$$

As the initial concentrations of B and Hf in this study are small, the activity coefficients of B in Al–Si melt and solid Si at the liquid and solid standard states are expressed with the first-order interaction parameters in Equations (14) and (15), respectively.

(14) $$\begin{array}{c} \text{ln}\gamma_{\text{B(l) in Al - Si melt}}=\text{ln}\gamma_{\text{B(l) in Al - Si melt}}^{\circ }+\varepsilon_{\text{B in Al - Si melt}}^{\text{B}}X_{\text{B in Al - Si melt}}^{}\\ \;+\varepsilon_{\text{B in Al - Si melt}}^{\text{Hf}}X_{\text{Hf in Al - Si melt}}^{} \end{array}$$

(15) $$\begin{array}{c} \text{ln}\gamma_{\text{B(s) in solid Si}}=\text{ln}\gamma_{\text{B(s) in solid Si}}^{\circ }+\varepsilon_{\text{B in solid Si}}^{\text{B}}X_{\text{B in solid S}}^{}+\varepsilon_{\text{B in solid Si}}^{\text{Al}}X_{\text{Al in solid Si}}^{}\\ \;+\varepsilon_{\text{B in solid Si}}^{\text{Hf}}X_{\text{Hf in solid Si}}^{} \end{array}$$

where $\varepsilon_{\text{B}}^{i}$ is interaction parameter of *i* on B in Al–Si melt or solid Si on mole fraction basis.

As the segregation ratios of Hf between solid Si and Al–Si melt is extremely small (estimated in this study and shown in Table 2), $X_{\text{Hf in solid Si}}\ll X_{\text{Hf in Al - Si melt}}$. The effect of Hf on $\gamma_{\text{B(s) in solid Si}}$ can be ignored. Therefore, the effect of Hf on $k_{\text{B}}$ can be attributed to $\varepsilon_{\text{B in Al - Si melt}}^{\text{Hf}}X_{\text{Hf in Al - Si melt}}^{}$ in Equation (14). Assuming the value of $\varepsilon_{\text{B in Al - Si melt}}^{\text{Hf}}$ is negative (because of the strong affinity between Hf and B), $k_{\text{B}}$ decreases with the increase of concentration of Hf according to Equations (13) and (14). Thus, more B would be released from solid Si and enriched in the Al–Si melt and at the Si grain boundaries before finally being stabilized as a B-containing compound, resulting in the improvement of B removal. This assumption agrees well with the experimental results obtained in this study (as shown in Figure 3), i.e. B could be removed more efficiently with larger initial concentration of Hf. Therefore, the enhancement of B removal by Hf addition can be ascribed to the negative value of $\varepsilon_{\text{B in Al - Si melt}}^{\text{Hf}}$ although its value has not been reported yet.

**Figure 6.**
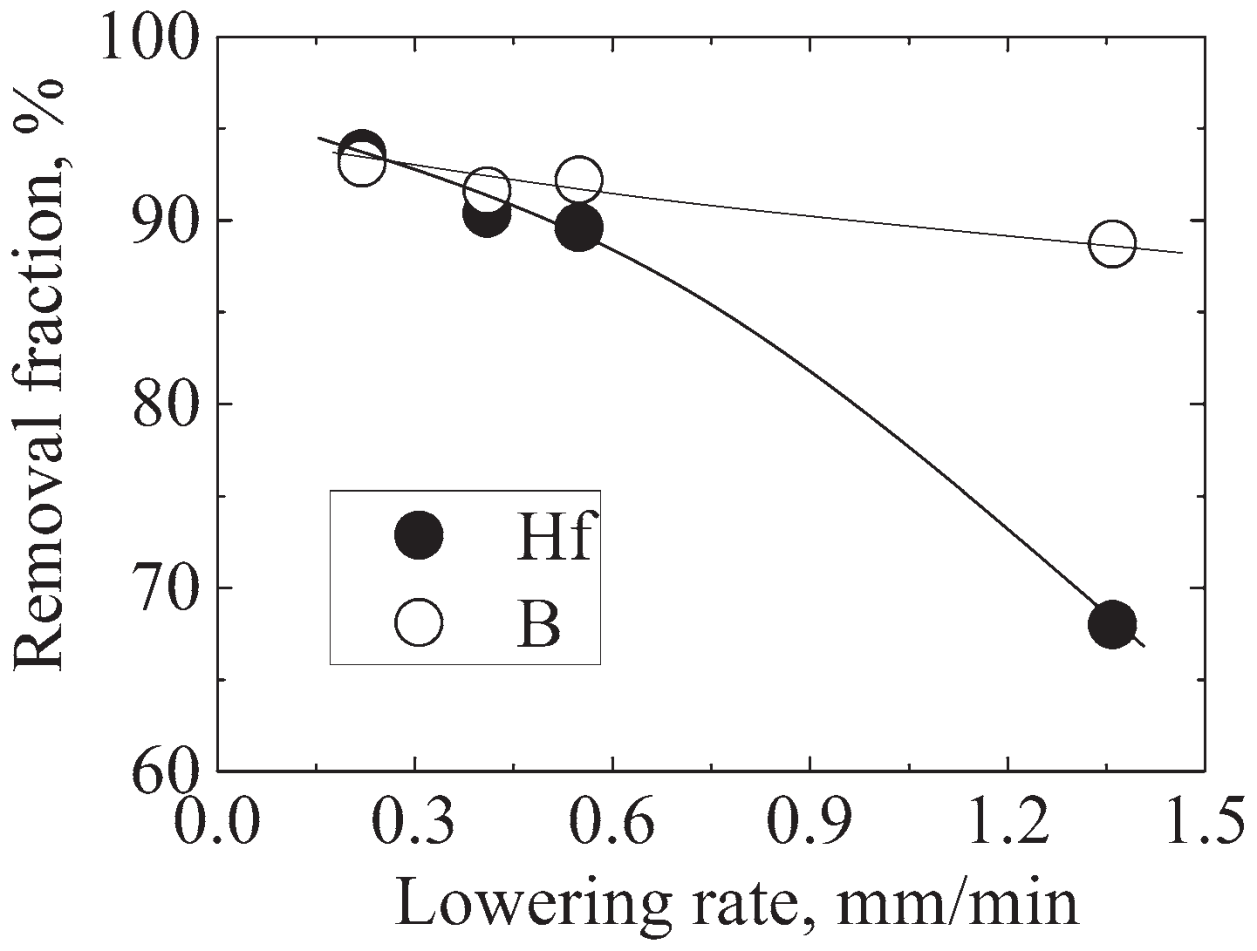
Removal fractions of B and Hf after electromagnetic solidification refinement of Si with varying lowering rates.

**Figure 7.**
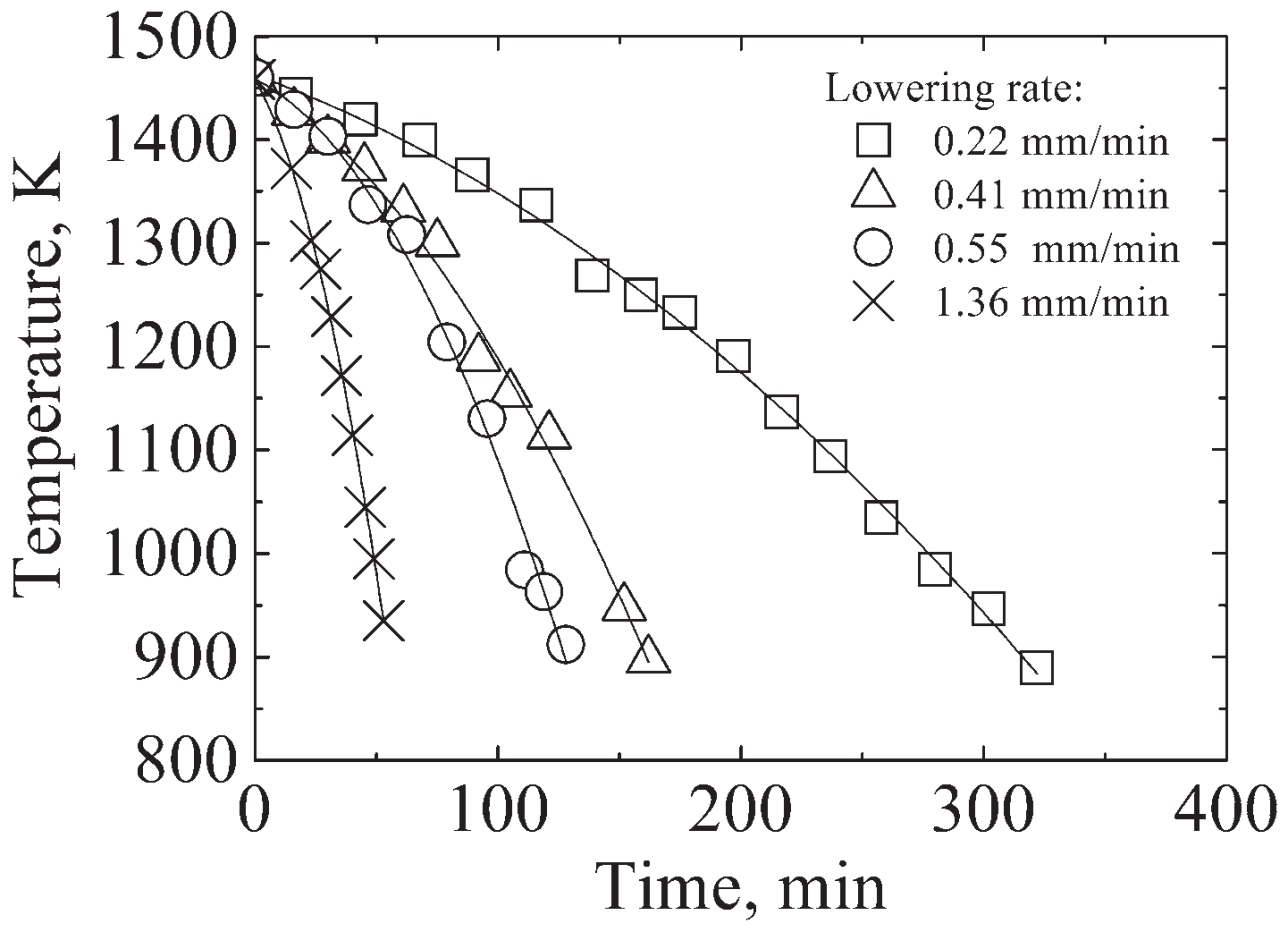
Temperature profiles during solidification with induction heating (Al–45 at.% Si alloy).

The amount of residual Hf in the refined Si remains significantly larger than its solubility in solid Si (less than 0.08 ppma below 1373 K [21]), as shown in Table 3. This finding indicates that the electromagnetic solidification refining process in this work is actually a non-equilibrium process. Kinetic factors, such as the cooling rate, should be necessarily considered. Figure 6 shows the effect of the lowering rate on B removal (Al–45 at.% Si was the refining solvent), and the corresponding temperature profiles are presented in Figure 7. The lowered rates employed in this study were 0.22, 0.41, 0.55, and 1.36 mm min^–1^. The corresponding cooling rates were 1.7–2.7, 3.4–5, 4.5–7.6, and 10.3–15.7 K min^–1^, respectively. According to Figure 6, the removal fractions of B and Hf increased from 88.7% to 93.1% and from 68.0% to 93.6%, respectively, with a decrease in the rate from 1.36 to 0.22 mm min^–1^, indicating that a slower cooling rate is more favorable for B and Hf removal. This result is expected because a slower cooling rate provides more time for the diffusion of B and Hf from the solid Si to the grain boundaries and liquid phases, thereby bringing the refining process much closer to its equilibrium.

**Figure 8.**
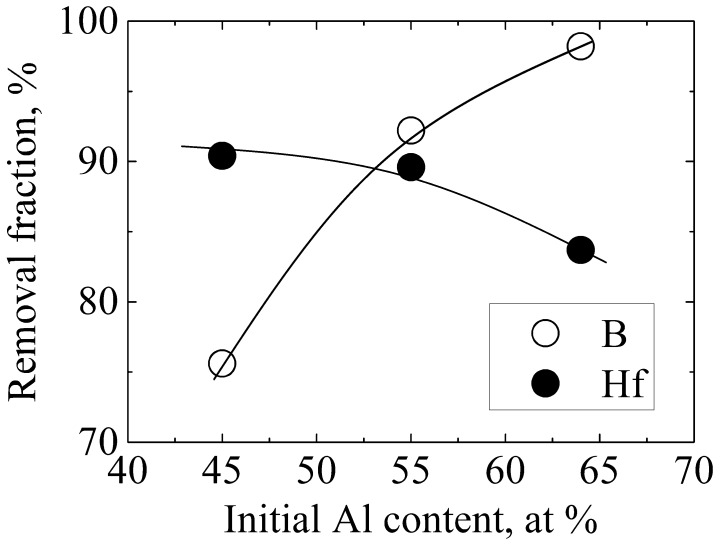
Removal fractions of B and Hf after electromagnetic solidification refinement of Si with varying initial Al content.

Figure 8 shows the effect of the melt composition on B removal at the lowered rate of 0.55 mm min^–1^. The liquidus temperatures of Al–55 at.% Si, Al–45 at.% Si, and Al–36 at.% Si alloys are 1373, 1273, and 1173 K, respectively. The removal fraction of B increases from 75.6% to 98.2%, and that of Hf slightly decreases from 90.4% to 83.7%, with the decrease of the liquidus temperature from 1373 K to 1173 K. The segregation ratio of B between solid Si and the Al–Si melt decreases from 0.8 to 0.22 with the decrease of the liquidus temperature of the Al–Si melt from 1687 K to 1273 K [9], which was responsible for the efficient B removal. However, according to Table 2, the segregation ratio of Hf also decreases with a decrease of the liquidus temperature of the Al–Si melt; Hf should thus be removed more efficiently when using a solvent with a lower liquidus temperature. The decrease of the removal fraction of Hf shown in Figure 8 was possibly due to the imperfect removal of Hf-containing compound by acid leaching.

As mentioned in section 1, Hf was expected to be more effective in removing B as it shows a stronger affinity for B than Ti does. However, the largest obtained removal fraction of B in this study was 98.2% (the B content decreased from 153 to 4.6 ppma) with the addition of 1025 ppma Hf (for the Al–45 at.% Si solvent). This value was slightly smaller than that reported in Yoshikawa’s study for the same refining solvent. In their study, the largest removal fraction of B was 99.4% (the B content decreased from 170 to 1.1 ppma) with the addition of 933 ppma Ti [15]. The difference may result from the different electromagnetic fields employed in the two studies (for example, 50 kHz in Yoshikawa’s study and 20 kHz in this study). For example, Ban et al. [13] confirmed that the electromagnetic field could significantly affect B removal.

To compare B removal by Hf addition to that by Ti addition under the same electromagnetic field, 1019 ppma Ti was added to the Al–45 at.% Si melt to investigate its effect on B removal. The experimental results are presented in Table 4. The results indicate that Hf is more effective in removing B than Ti in the same electromagnetic solidification refining process.

**Table 4.** Experimental results of B removal by Hf addition compared with those with Ti addition.

	Cooling rate, K min^–1^	Initial composition of Al-45 at.% Si melt, ppma			Final composition of refined Si, ppma			
		B	Hf	Ti	B	Hf	Ti	Al
This study	4.5 to 7.6	153	1025	—	4.6	127	—	435
	4.5 to 7.6	153	—	1019	37.1	—	248	745
[15]	5 to 10	170	—	933	1.1	—	1.6	726

### 3.3. Proposal of the SoG-Si production route from Al–Si solvent refining with Hf addition

**Figure 9.**
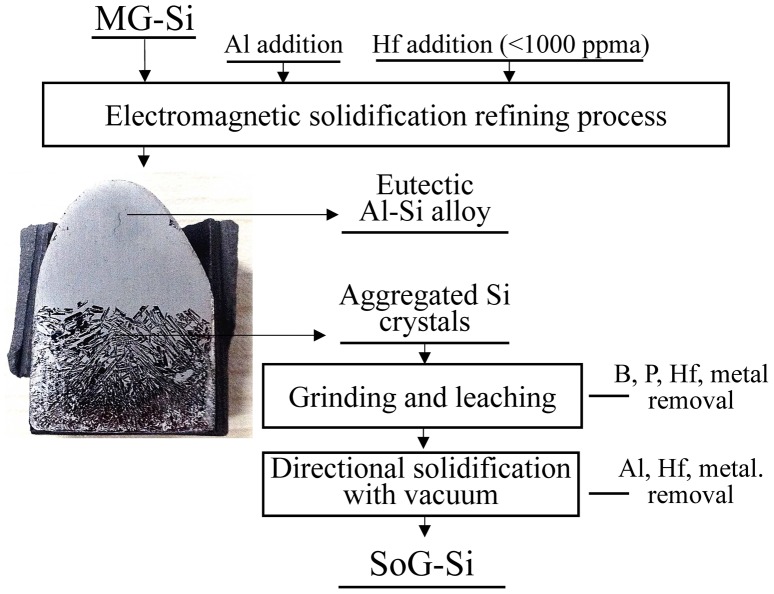
The overall process for producing SoG-Si from MG-Si using Al–Si solvent with Hf addition. (The removal of P using Al–Si solvent has been reported previously [26]).

The combination of Al–Si solvent refining and Hf addition can allow more effective removal of B. Based on the results obtained in this study, B removal will be more efficient with the addition of more Hf and the use of a slower cooling rate combined with a lower liquidus temperature of the Al–Si melt as the refining solvent. Although residual Al and Hf are present in the refined Si, they are expected to be removed under a vacuum directional-solidification refining process because the vapor pressure of Al is higher than that of Si and the segregation coefficient of Hf is very small. Consequently, the overall process for producing SoG-Si from MG-Si can be proposed and is illustrated in Figure 9. The efficient removal of P using an Al–Si solvent has been confirmed by Yoshikawa and Morita [26].

## 4. Conclusions

A small amount of Hf was used as a new additive to improve B removal in the electromagnetic solidification refinement of Si with an Al–Si solvent because of its strong affinity for B. The segregation ratio of Hf between solid Si and the Al–Si melt was estimated to be very small. With the obtained segregation ratio, the activity coefficient of Hf in solid Si at its infinite dilution relative to pure solid Hf was estimated to be:

$$RT\ln\gamma_{\text{Hf (s) in solid Si}}^{\circ }=61110\left(\pm 16200\right)-30\left(\pm 11\right)T\left(J/mol\right)$$

The B removal could be significantly improved with a small Hf addition and was more efficient at a slower cooling rate combined with a lower liquidus temperature of the Al–Si melt as the refining solvent. The maximum removal fraction of B was 98.2% with 410 ppma Hf addition when the liquidus temperature of the Al–Si melt was 1173 K and the cooling rate was 4.5–7.6 K min^–1^. Finally, the overall process for producing SoG-Si from MG-Si using an Al–Si solvent with Hf addition was proposed.

## Funding

This study was sponsored by the National Natural Science Foundation of China [grant numbers 51504118, 51574133].

## Disclosure statement

No potential conflict of interest was reported by the authors.
